# Dry Eye after Small Incision Lenticule Extraction (SMILE) versus Femtosecond Laser-Assisted in Situ Keratomileusis (FS-LASIK) for Myopia: A Meta-Analysis

**DOI:** 10.1371/journal.pone.0168081

**Published:** 2016-12-16

**Authors:** Zeren Shen, Yanan Zhu, Xiaohui Song, Jie Yan, Ke Yao

**Affiliations:** Eye Center, Second Affiliated Hospital, School of Medicine, Zhejiang University, Hangzhou, China; Xiamen University, CHINA

## Abstract

**Purpose:**

To compare dry eye after small incision lenticule extraction (SMILE) and femtosecond laser-assisted LASIK (FS-LASIK) for correcting myopia.

**Methods:**

CENTRAL, Embase and PubMed were searched in November 2016. All randomized controlled trials (RCTs) and prospective cohorts that compared dry eye after SMILE with FS-LASIK were selected.

**Results:**

Five cohorts and one RCT were identified for comparing dry eye after SMILE (291 eyes) and FS-LASIK (277 eyes). The pooled results revealed that the SMILE and FS-LASIK groups did not differ significantly in terms of Schirmer’s I test (SIT) and tear film osmolarity (TFO) at any postoperative visits. By contrast, tear break up time (TBUT; *p* = 0.04 for one month, *p* < 0.001 for three months, and *p* = 0.02 for six months) and ocular surface disease index (OSDI; *p* < 0.001 for one month and three months, and *p* = 0.006 for six months) were significantly worse in the FS-LASIK group than in the SMILE group at follow-up. At six months postoperatively, TBUT and TFO values in both the SMILE and FS-LASIK groups and OSDI scores in the SMILE group returned to preoperative levels, but SIT values in both groups (*p* = 0.02 for the SMILE group and *p* < 0.001 for the FS-LASIK group) and OSDI in the FS-LASIK group (*p* < 0.001) were still statistically impaired.

**Conclusion:**

Dry eye after both SMILE and FS-LASIK usually occurs transiently. SMILE does not show obvious superiority over FS-LASIK by exhibiting similar and acceptable objective parameters, and SMILE may have milder subjective symptoms.

## Introduction

Refractive surgery, such as laser-assisted in situ keratomileusis (LASIK), allows people to reduce their dependence on glasses. Although high satisfaction is reported, dry eye remains the most common complication after LASIK; the incidence varies among patients [1–5]. Some patients experienced transient dry eye, while others reported severe symptoms over the long term, with incidence ranging from 20% to 40% [2]. It is thus clear that a significant number of patients are at risk for developing chronic dry eye disease, further affecting the health status of this robust population [6].

Total amputation of the corneal nerves due to flap creation and photoablation is a likely cause of post-LASIK dry eye [2,7]. Traditionally, the flap is created using mechanical microkeratomes, but femtosecond laser technology has become increasingly common [8]. Femtosecond laser-assisted LASIK (FS-LASIK) generates more consistent and predictable flap diameters, thicknesses, and hinge widths than microkeratomes [9]. The control and optimization of corneal features may reduce flap-related complications such as reduced corneal nerve injury and encourage faster recovery from dry eye [9–11].

With the introduction of the femtosecond laser platform (VisuMax, Carl Zeiss Meditec AG, Jena, Germany), small incision lenticule extraction (SMILE) emerged as a novel, all-in-one refractive surgery for myopia. It is a flapless procedure in which an intrastromal lenticule is created between two photodisruption planes and removed mechanically from an arcuate side cut of 3 to 4 mm [12], which is much shorter than the length of a standard LASIK flap. This minimally invasive approach was intended to preserve corneal nerves more successfully and result in lower incidence of dry eye than found with FS-LASIK and traditional LASIK [13].

Recent studies have compared dry eye after SMILE and FS-LASIK [6,12,14–17]. Some studies supported the position that SMILE reduced the incidence of dry eye disease when compared with FS-LASIK [6,14,15,17], but others reported no differences in dry eye parameters between these two groups [12,16]. One meta-analysis cited dry eye as a primary outcome when comparing SMILE and FS-LASIK [5]. Only two to four studies were included in each comparison of this meta-analysis [6,12,14,15], and the comparisons and follow-up durations were neither adequate nor complete. Uncertainty remains because the results are controversial and the sample sizes remain very small. Thus, a meta-analysis was performed to compare dry eye after SMILE versus FS-LASIK at different follow-up periods.

## Materials and Methods

### Search strategy

The systematic review and meta-analysis was performed in accordance with PRISMA guidelines. The PubMed, Embase, and Cochrane Central Register of Controlled Trials (CENTRAL) were searched independently by two reviewers for records that compared dry eye after SMILE and LASIK. The search terms were related to LASIK (e.g., ‘Keratomileusis, Laser In Situ’ and ‘LASIK’) and SMILE (e.g., ‘lenticule extraction’ and ‘SMILE’). S1 Appendix shows the PubMed search process in detail. No date restrictions were applied in the electronic search for trials; the last search was run on November 14, 2016. The search was limited to English-language papers. Titles and abstracts were independently screened by two reviewers, after which potentially relevant reports were retrieved as complete manuscripts and assessed for compliance with inclusion criteria. Discrepancies between the reviewers were resolved by discussion.

### Inclusion criteria and exclusion criteria

The following selection criteria were used to identify studies for inclusion in this meta-analysis: 1) study design: randomized controlled trials (RCTs) and cohort studies; 2) population: participants with stable myopia or myopic astigmatism and without ocular diseases, especially dry eye disease; 3) intervention: SMILE versus FS-LASIK, the use of standard surgical techniques. SMILE was performed using a femtosecond laser (VisuMax) with a 100–120 μm thick cap and a 6.0–6.6 mm diameter lenticule. For the FS-LASIK group, the corneal flap was made by a femtosecond laser with a 90–110 μm thick and 7.9–9.0 mm diameter flap, and a 50° superior hinge. Excimer photoablation was performed in a 6.0–6.5 mm optical zone; 4) outcome: dry eye parameters. Letters, review articles, animal or laboratory studies, and conference abstracts were all excluded.

### Outcome measures

The outcome measures for inclusion were ocular surface disease index (OSDI), tear breakup time (TBUT), Schirmer’s I test (SIT), and tear film osmolarity (TFO) at one week, one month, three months, and six months postoperatively; at least one of the outcome measures was required for inclusion.

### Data collection and quality assessment

Two reviewers independently collected the data and assessed the quality of studies. Any disagreements between the reviewers’ results were resolved by discussion that involved a third reviewer when necessary. The following information was extracted from each study: first author, year of publication, study design, location, age of patients, number of eyes enrolled, preoperative spherical equivalent, preoperative dry eye parameters (OSDI, TBUT, SIT, and TFO), surgical procedures, follow-up duration, and outcome data. Studies with incomplete data were also included; authors were contacted to provide sufficient information when necessary. We contacted four authors, and one responded [6].

The quality of RCTs was assessed using the Jadad Scale [18], while the Newcastle-Ottawa Scale (NOS) was adopted to evaluate each cohort [19]. The Jadad scale features three principal assessment domains: randomization, blinding, and participant dropout. Appropriate randomization and blinding scored two points each, and total scores ranged from zero to five. Studies scoring fewer than three points were considered to be of low quality. The maximum NOS score is nine, based on assessing three areas: selection quality (maximum four points), comparability (maximum two points), and outcome measures (maximum three points). Studies scoring five or fewer points were considered to be of low quality [3].

### Statistical analysis

RevMan software (version 5.3; Cochrane Collaboration, Oxford, United Kingdom) was used to analyze the data. The mean difference (MD) and corresponding 95% confidence interval (CI) were calculated for continuous outcomes; *p* < 0.05 was considered statistically significant.

Statistical heterogeneity was tested using the chi-square-based Q-test and the I^2^ statistic. *I*^*2*^ > 50% and *p* < 0.1 for the Q-test indicated significant heterogeneity, so the random effects model was used in those cases. Otherwise, the fixed effects model was used [20].

A sensitivity analysis was performed to evaluate the robustness of the results. In a leave-one-out cross-validation, each study in the meta-analysis was excluded in turn to investigate the influence of individual studies on the pooled estimates [21]. Publication bias was measured using a Begg funnel plot [22].

## Results

### Search results

**Fig 1:**
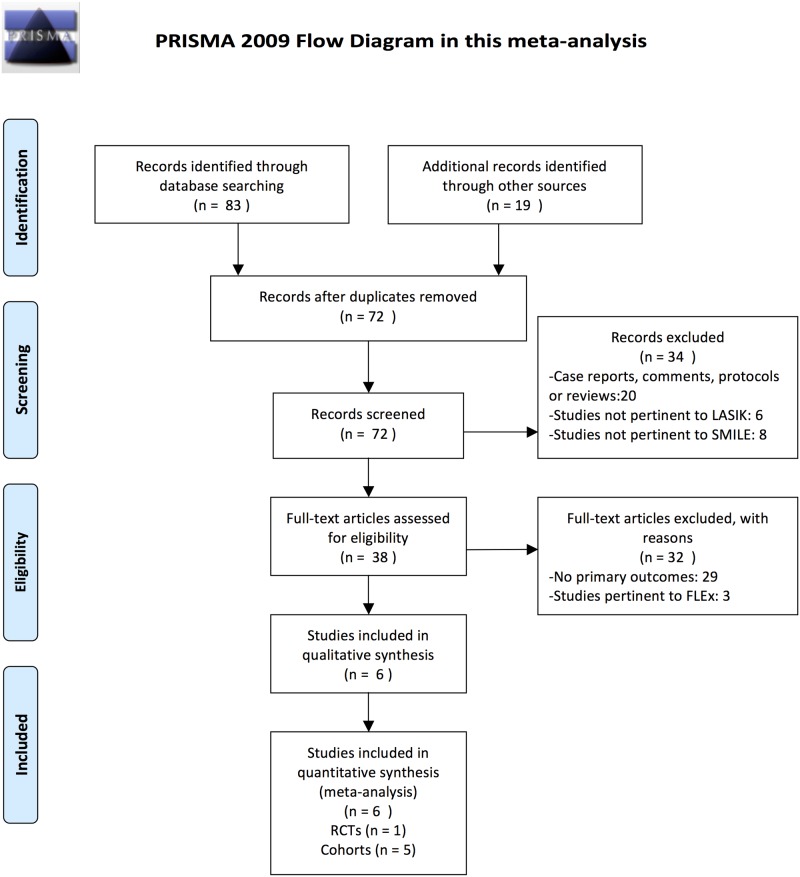
Flow chart showing literature search strategy. LASIK = laser in situ keratomileusis; SMILE = small incision lenticule extraction; FLEx = femtosecond lenticule extraction; RCTs = randomized controlled trials. *From*: Moher D, Liberati A, Tetzlaff J, Altman DG, The PRISMA Group (2009). *P*referred *R*eporting *I*tems for *S*ystematic Reviews and *M*eta-*A*nalyses: The PRISMA Statement. PloS Med 6(7): e1000097. doi: 10.1371/journal.pmed1000097.

The search found 72 citations, 34 of which were excluded by the initial search and screening of titles and abstracts. After further consideration of the remaining 38, we excluded 32 studies for following reasons: 29 did not provide the primary outcomes required for this meta-analysis, and three were not pertinent to SMILE or LASIK procedures. Five prospective cohort studies and one RCT were included in the final meta-analysis [6,12,14–17]. Fig 1 is a flow diagram detailing the search and selection process.

### Study characteristics and quality

Six studies that reported on 291 eyes in the SMILE group and 277 eyes in the FS-LASIK group provided data for our meta-analysis. Four studies were conducted in China [14–17], one in Turkey [12], and one in France [6]. The six studies’ main characteristics are described in Table 1, and their quality is assessed in Tables 2 and 3. The RCT scored only two because masking of surgeons is impossible and masking of patients was not reported [12]. The quality of the included cohorts was assessed by the NOS [6,14–17]. No selection bias was found in any of the studies. For comparability, most important factors were controlled in all studies. For outcome, one study had a twelve-month follow-up [16]. Total scores in all of the cohorts were above five, indicating a low risk of bias.

**Table 1:** Characteristics of Studies Included in the Meta-Analysis Comparing the Small Incision Lenticule Extraction (SMILE) and Femtosecond Laser-Assisted Laser in Situ Keratomileusis (FS-LASIK).

Study	Year	Design	Location	SMILE				FS-LASIK				Follow-up (m)
				Eyes (n)	Age (y)	Preoperative SE (D)	Surgical Procedure	Eyes (n)	Age (y)	Preoperative SE (D)	Surgical Procedure	
Demirok et al[12]	2013	Randomized	Turkey	28	26.2 ± 4.4	-4.0 ± 1.4	VisuMax FS	28	26.2 ± 4.4*	-3.9 ± 1.5	VisuMax FS and Schwind Amaris excimer laser	6
Denoyer et al[6]	2015	Cohort (prospective)	France	30	31.1 ± 4.7	-4.65 ± 2.38	VisuMax FS	30	32.2 ± 7.5	-4.42 ± 1.78	IntraLase FS and Alleggretto excimer laser	6
Li et al[14]	2013	Cohort (prospective)	China	36	28.21 ± 7.04	-6.68 ± 1.34	VisuMax FS	30	27.33 ± 6.58	-7.96 ± 2.61	VisuMax FS and MEL-80 excimer laser	6
Wang et al[16]	2015	Cohort (prospective)	China	47	25.21 ± 6.51	-7.46 ± 1.11	VisuMax FS	43	24.72 ± 6.53	-7.44 ± 1.13	VisuMax FS and MEL-80 excimer laser	12
Xia et al[17]	2016	Cohort (prospective)	China	69	25.15 ± 4.42	-5.04 ± 2.32	VisuMax FS	59	23.65 ± 3.87	-5.13 ± 1.36	VisuMax FS and Alleggretto excimer laser	6
Xu et al[15]	2014	Cohort (prospective)	China	81	24.10 ± 6.03	-5.70 ± 1.71	VisuMax FS	97	23.96 ± 5.14	-5.80 ± 2.01	VisuMax FS and MEL-80 excimer laser	6

SE, spherical equivalent; D, diopter; m, month; y, year.

* The mean age of SMILE and FS-LASIK groups, no separate data provided.

**Table 2:** Jadad Scale for Randomized Controlled Trials (RCTs).

Study	Randomization	Blinding	Withdraws	Sum of Score
Demirok et al[12] 2013	1	0	1	2

**Table 3:** Newcastle–Ottawa Scale for Observational Studies (cohorts).

Study	Selection	Comparability	Outcome	Total score
Denoyer et al[6] 2015	****	**	**	8
Li et al[14] 2013	****	*	**	7
Wang et al[16] 2015	****	**	***	9
Xia et al[17] 2016	****	**	**	8
Xu et al[15] 2014	****	*	**	7

A higher overall score corresponds to a lower risk of bias; a score of five or less (out of nine) indicates a high risk of bias. Each * equals 1 point.

### Outcome criteria

#### TBUT

**Fig 2:**
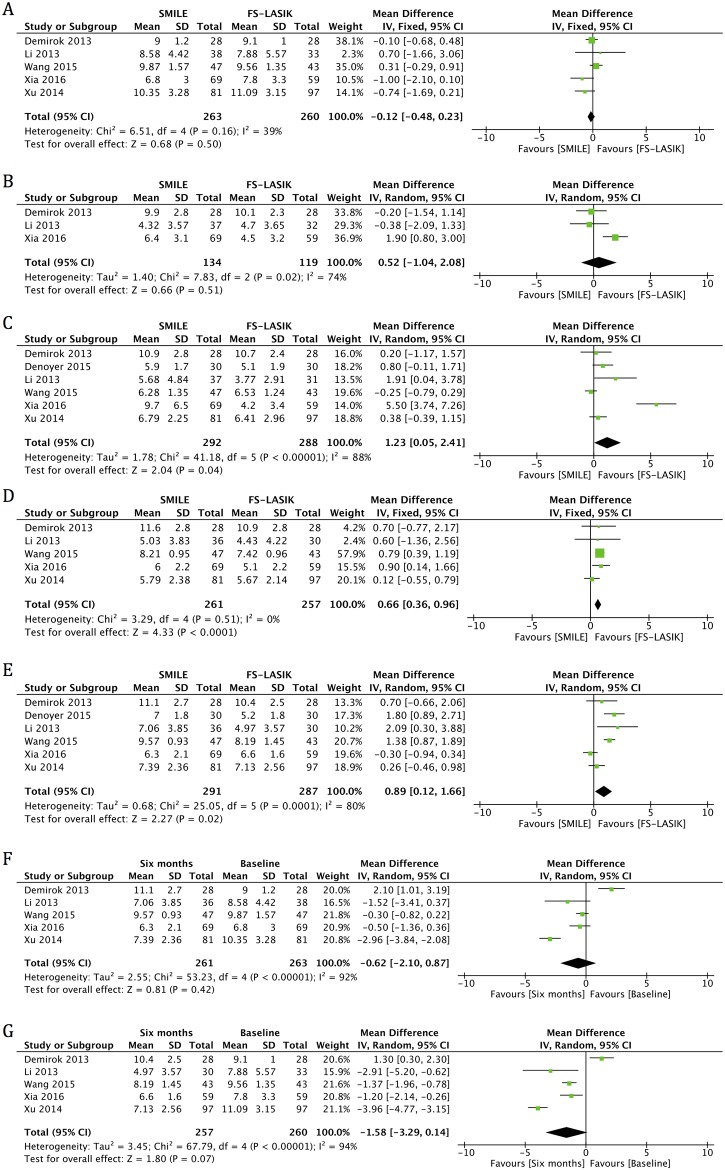
Forest plot showing the mean difference (MD) of tear breakup time (TBUT) comparing small incision lenticule extraction (SMILE) with femtosecond laser-assisted laser in situ keratomileusis (FS-LASIK) (A) preoperatively and in postoperative visits at (B) one week, (C) one month, (D) three months and (E) six months. Forest plot showing the MD of TBUT in the (F) SMILE and (G) FS-LASIK groups at six months postoperatively compared with preoperatively. CI = confidence interval; SD = standard deviation.

All studies reported TBUT at one month and six months postoperatively [6,12,14–17]. An examination of the forest plot showed no statistically significant change in either the SMILE and FS-LASIK groups at six months (MD -0.62, 95% CI: -2.10 to 0.87, *p* = 0.42; MD -1.58, 95% CI: -3.29 to 0.14, *p* = 0.07) (Fig 2F and 2G, Table 4) postoperatively compared with preoperatively. Significantly higher TBUT scores were found in the SMILE group than in the LASIK group at all follow-up visits (MD 1.23, 95% CI: 0.05 to 2.41, *p* = 0.04 for one month; MD 0.66, 95% CI: 0.36 to 0.96, *p* < 0.001 for three months; and MD 0.89, 95% CI: 0.12 to 1.66, *p* = 0.02 for six months), except for one week after surgery (MD 0.52, 95% CI: -1.04 to 2.08, *p* = 0.51) (Fig 2B–2E, Table 4).

**Table 4:** Results of the examination of the forest plots (*p* value).

	Six months versus Baseline (*p* value)		SMILE versus FS-LASIK (*p* value)				
	The SMILE group	The FS-LASIK group	Baseline	One week post-surgery	One month post-surgery	Three months post-surgery	Six months post-surgery
**TBUT**	0.42	0.07	0.50	0.51	^†^0.04	^†^< 0.0001	^†^0.02
**SIT**	^†^0.02	^†^< 0.00001	0.42	0.20	0.57	0.19	0.62
**OSDI**	0.79	^†^< 0.00001	0.53	–	^†^< 0.00001	^†^< 0.00001	^†^0.006
**TFO**	–	–	–	–	0.41	–	0.46

TBUT, tear breakup time; SIT, Schirmer’s I test; OSDI, ocular surface disease index; TFO, tear film osmolarity.

^†^*p* < 0.05.

#### SIT

**Fig 3:**
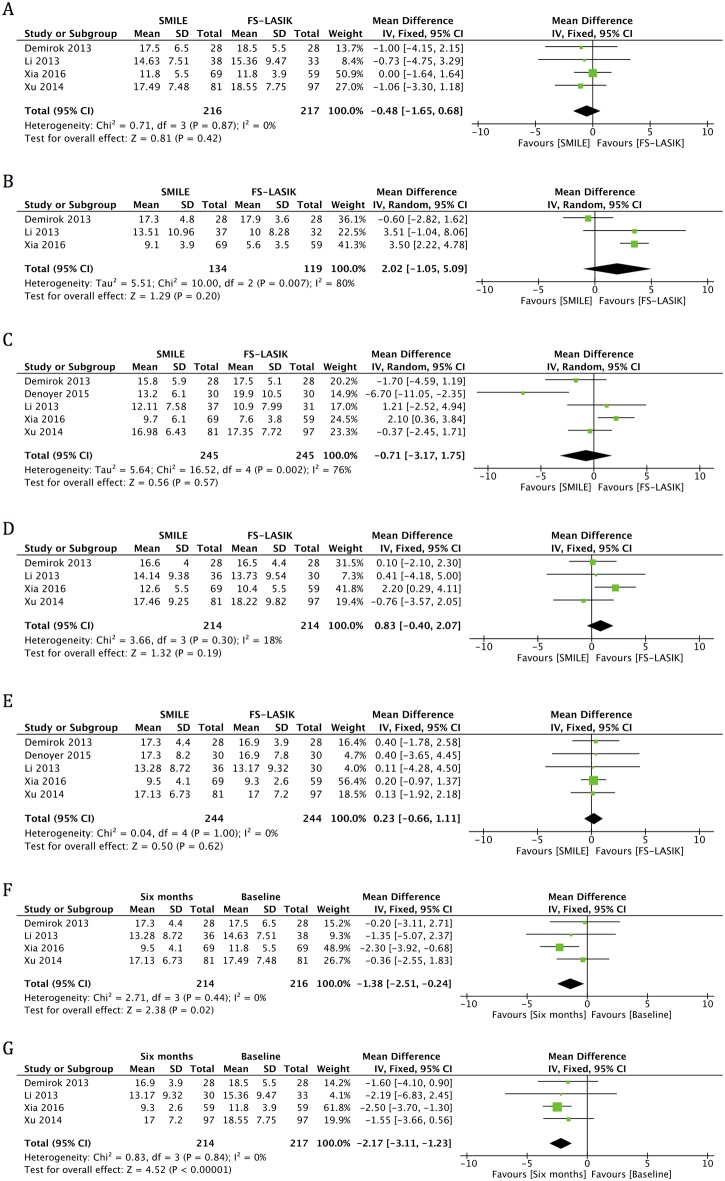
Forest plot showing the mean difference (MD) on the Schirmer’s I test (SIT) comparing small incision lenticule extraction (SMILE) with femtosecond laser-assisted laser in situ keratomileusis (FS-LASIK) (A) preoperatively and in postoperative visits at (B) one week, (C) one month, (D) three months and (E) six months. Forest plot showing the MD of SIT in the (F) SMILE and (G) FS-LASIK groups at six months postoperatively compared with preoperatively. CI = confidence interval; SD = standard deviation.

Five studies reported data for SIT scores at one month and six months postoperatively [6,12,14,15,17]. An examination of the forest plot demonstrated that statistically significant decreases in the SIT in both SMILE and FS-LASIK groups were found at six months postoperatively, compared with preoperative values (MD -1.38, 95% CI: -2.51 to -0.24, *p* = 0.02; MD -2.17, 95% CI: -3.11 to -1.23, *p* < 0.001) (Fig 3F and 3G, Table 4). No significant difference was found between the two groups in postoperative visits at one week (MD 2.02, 95% CI: -1.05 to 5.09, *p* = 0.20), one month (MD -0.71, 95% CI: -3.17 to 1.75, *p* = 0.57), three months (MD 0.83, 95% CI: -0.40 to 2.07, *p* = 0.19), and six months (MD 0.23, 95% CI: -0.66 to 1.11, *p* = 0.62) (Fig 3B–3E, Table 4).

#### OSDI

**Fig 4:**
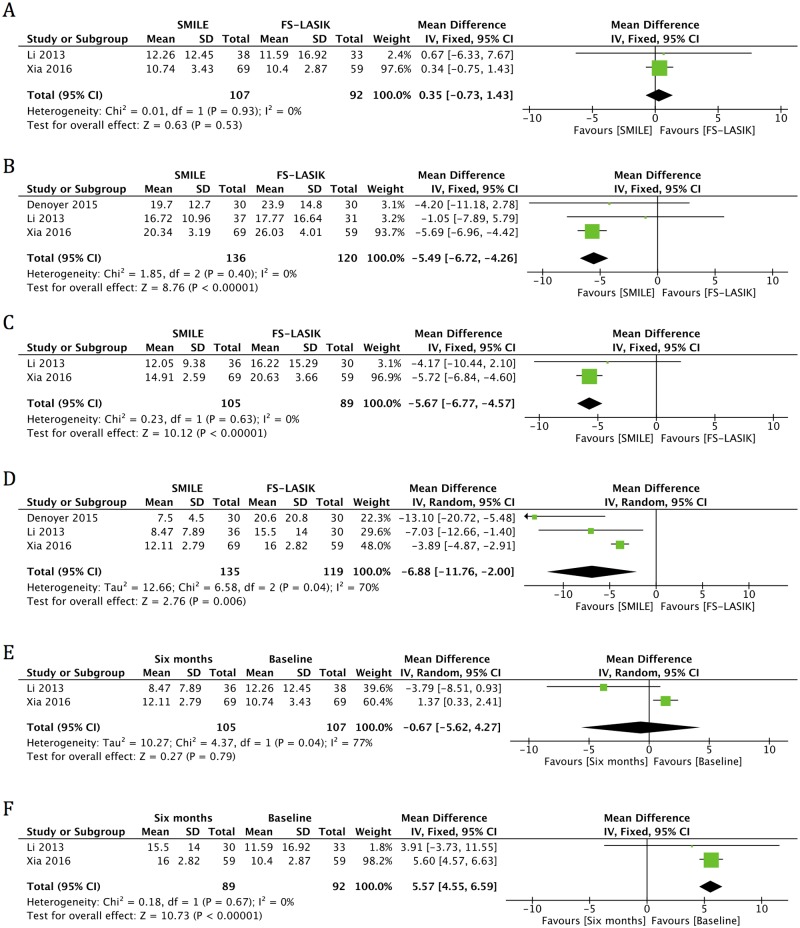
Forest plot showing the mean difference (MD) in ocular surface disease index (OSDI) comparing small incision lenticule extraction (SMILE) with femtosecond laser-assisted laser in situ keratomileusis (FS-LASIK) (A) preoperatively and in postoperative visits at (B) one month, (C) three months and (D) six months. Forest plot showing the MD of OSDI in the (E) SMILE and (F) FS-LASIK groups at six months postoperatively compared with preoperatively. CI = confidence interval; SD = standard deviation.

Three publications reported OSDI at one month and six months postoperatively [6,14,17]. An examination of the forest plot revealed significant increases in postoperative OSDI scores in the FS-LASIK group at six months (MD 5.57, 95% CI: 4.55 to 6.59, *p* < 0.001), but scores in the SMILE group returned to preoperative levels at six months (MD -0.67, 95% CI: -5.62 to 4.27, *p* = 0.79) (Fig 4E and 4F, Table 4). In addition, there was a significantly lower OSDI score in the SMILE group than in the FS-LASIK group at all time points (MD -5.49, 95% CI: -6.72 to -4.26, *p* < 0.001 for one month; MD -5.67, 95% CI: -6.77 to -4.57, *p* < 0.001 for three months; MD -6.88, 95% CI: -11.76 to -2.00, *p* = 0.006 for six months) (Fig 4B–4D, Table 4).

#### TFO

**Fig 5:**
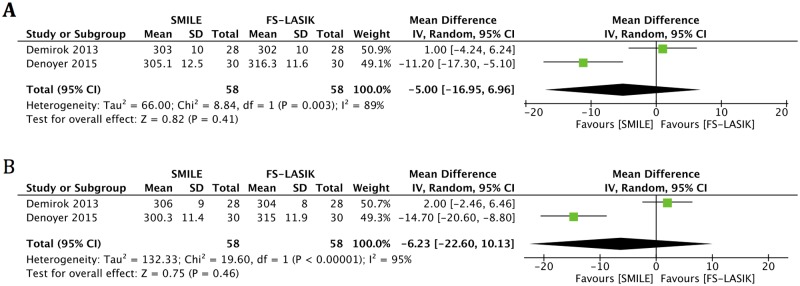
Forest plot showing the mean difference (MD) in tear film osmolarity (TFO) comparing small incision lenticule extraction (SMILE) with femtosecond laser-assisted laser in situ keratomileusis (FS-LASIK) at (A) one month and (B) six months postoperatively. CI = confidence interval; SD = standard deviation.

Two publications reported data for TFO [6,12]. There was no statistically significant change in either group at any time point postoperatively compared with preoperatively [12]. An examination of the forest plot revealed no significant differences between the two groups at one month (MD -5.00, 95% CI: -16.95 to 6.96, *p* = 0.41) and six months (MD -6.23, 95% CI: -22.60 to 10.13, *p* = 0.46) after surgery (Fig 5A and 5B, Table 4).

### Sensitivity analysis and publication bias

The results of the leave-one-out analysis on the majority of outcomes showed that most exclusions did not alter the results of the previous analyses. After excluding Xia et al.’s study [17], heterogeneity was reduced in TBUT outcome (I^2^ from 74% to 0% for one week post-surgery and I^2^ from 88% to 49% for one month post-surgery) and OSDI outcome (I^2^ from 70% to 37% for six months post-surgery), but the pooled results remained unchanged. In the SIT outcome, heterogeneity was reduced (I^2^ from 80% to 0% for one week post-surgery) after excluding the study by Demirok et al. [12], and the pooled result showed that SIT scores were significantly higher in the SMILE group than in the FS-LASIK group in the remaining studies. No significant publication bias was demonstrated in the funnel plot.

## Discussion

Given the popularity of refractive surgery and the prevalence of ocular dryness complaints after such procedures, we performed a meta-analysis to compare dry eye after SMILE versus FS-LASIK at one week, one month, three months, and six months postoperatively. According to the Dry Eye Workshop definition, dry eye disease is a multifactorial pathology that includes tear film changes with or without corneal damage, ocular symptoms, visual degradation, and decreased tear osmolarity, which together degrade quality of life [6]. Thus, a full and appropriate evaluation composed of tear film quality (as measured by TBUT and TFO), patient-reported, vision-related quality of life (as measured by OSDI), and the quantity of tear fluid (as measured by SIT) was adopted.

Previous meta-analyses suggested that SMILE shows obvious superiority over FS-LASIK by exhibiting a lower risk of postoperative dry eye [3–5]. However, a different conclusion has been drawn in our study.

In terms of tear film quality, although the SMILE group had a longer TBUT at one month, three months, and six months postoperatively than the FS-LASIK group, there were no statistically significant changes in TBUT and TFO values at six months postoperatively compared with preoperatively in either group. In terms of fluid quantity, SIT values were significantly decreased at the six-month postoperative visit in both SMILE and FS-LASIK groups. There was no significant difference between these two groups at any time point. Denoyer et al.’s provided the postoperative values at six months but could not provide preoperative values [6], so the number of studies included in the comparison for FS-LASIK versus SMILE at six months postoperatively and the comparisons for postoperative six months versus preoperative values in both FS-LASIK and SMILE groups are different, which explains the different results in these comparisons. In general, both SMILE and FS-LASIK achieved acceptable tear film quality and similarly decreased tear fluid quantity at six months postoperatively. The SMILE group does not have obvious competitive superiority in objective parameters over the FS-LASIK group.

Subjective symptoms are also important in the diagnosis of dry eye. The various studies used different questionnaires to compare subjective symptoms. There was no significant difference between the SMILE and FS-LASIK groups in either the McMonnies score [15] or the Salisbury eye evaluation questionnaire [16] at the end of follow-up. The OSDI questions were drawn from three subscales: ocular symptoms, vision-related functions, and environmental triggers [14]. In our analysis, significant differences in OSDI scores between the SMILE and FS-LASIK groups existed at all time points. Moreover, OSDI scores in the SMILE group returned to preoperative levels at six months, but significant increases in the postoperative scores were still found in the FS-LASIK group at that same point in time. It thus appears that people in the SMILE groups enjoyed significantly better vision-targeted, health-related quality of life. Considering the two studies [14,17] that reported OSDI scores employed cohorts without blinding methods, psychological factors may have influenced the accuracy of and confidence in the results, meaning that some people could have preferred SMILE because it was a new and ostensibly better approach.

Like dry eye, corneal sensation reduction is fairly common after refractive surgery. The included RCT suggested that there was no association between corneal sensation and dry eye parameters [12], but other studies have suggested that decreased corneal sensation does play a role in postoperative dry eye [2,6,23–25]; one possible explanation is that corneal nerves can be cut during flap creation in LASIK, and subsequent excimer ablation further severs stromal nerve fiber bundles, leading to decreased corneal sensation and increased dry eye symptoms [26]. The SMILE procedure, by contrast, uses a small side cut instead of creating a flap and achieves refractive change by lenticule creation with a femtosecond laser instead of by photoablation with an excimer laser. One meta-analysis reported that corneal sensitivity in the SMILE group recovered faster than in the FS-LASIK group during the first three months postoperatively, but that recovery was similar six months after surgery [13].

Our results should be interpreted with caution, because the study has a relatively small sample size, given the low number of published articles. Furthermore, we conducted a sensitivity analysis by excluding each study in turn to investigate the influence of the individual studies on the pooled estimates. While that analysis did not alter most primary analysis results, it did reveal that Xia et al.’s [17] and Demirok et al.’s [12] studies were the major source of statistical heterogeneity for TBUT and SIT respectively. This heterogeneity may have been due to the use of artificial tears in the SMILE group in Xia et al.’s study and design differences between Demirok et al.’s study and the others. Heterogeneity may also arise due to regional origin, changes in technology, and other factors, but that could not be explored formally because of the low number of included studies. A random effects model was used in outcomes with statistical heterogeneity to obtain a relatively conservative result.

Quality assessment showed that all included cohorts had good quality, although the RCT scored relatively poorly. Trials have found that three to six months are needed for corneal nerves to return to preoperative status after refractive surgery [27–30]. The follow-up visits in each included study went on for at least six months, which makes our conclusion practical and could help patients make more informed decisions.

In conclusion, the present meta-analysis suggests that dry eye after both SMILE and FS-LASIK usually occurs transiently. SMILE does not show obvious superiority over FS-LASIK by exhibiting similar and acceptable objective parameters, and SMILE may have milder subjective symptoms.

## Supporting Information

S1 Appendix Search strategy of PubMed. (DOCX)

S2 Appendix PRISMA-checklist in this meta-analysis. (DOC)
