# Supplementary material for: Dry Eye after Small Incision Lenticule Extraction (SMILE) versus Femtosecond Laser-Assisted in Situ Keratomileusis (FS-LASIK) for Myopia: A Meta-Analysis
Source: PLoS One. 2016 Dec 16;11(12):e0168081. doi: 10.1371/journal.pone.0168081 (PMC5161410; doi:10.1371/journal.pone.0168081)
Supplement: S1 Appendix — (DOCX) [file pone.0168081.s001.docx]

S1 Appendix. Medline (PubMed) search strategy

#1 Dry Eye Syndromes[Mesh]

#2 dry eye[tiab]

#3 xerophthalmia[tiab]

#4 Sjogren*[tiab]

#5 #1 OR #2 OR #3 OR #4

#6 Keratomileusis, Laser In Situ[Mesh]

#7 Keratomileus*[tiab]

#8 LASIK [tiab]

#9 #6 OR #7 OR #8

#10 lenticule extraction[tiab]

#11 ReLEx[tiab]

#12 SMILE[tiab]

#13 #10 OR #11 OR #12

#14 #5 AND #9 AND #13
